# Supplementary material for: Asymmetric ephaptic inhibition between compartmentalized olfactory receptor neurons
Source: Nat Commun. 2019 Apr 5;10:1560. doi: 10.1038/s41467-019-09346-z (PMC6451019; doi:10.1038/s41467-019-09346-z)
Supplement: Supplementary file 1 — Supplementary Information [file 41467_2019_9346_MOESM1_ESM.pdf]

Asymmetric ephaptic inhibition between compartmentalized olfactory receptor neurons

Zhang et al.

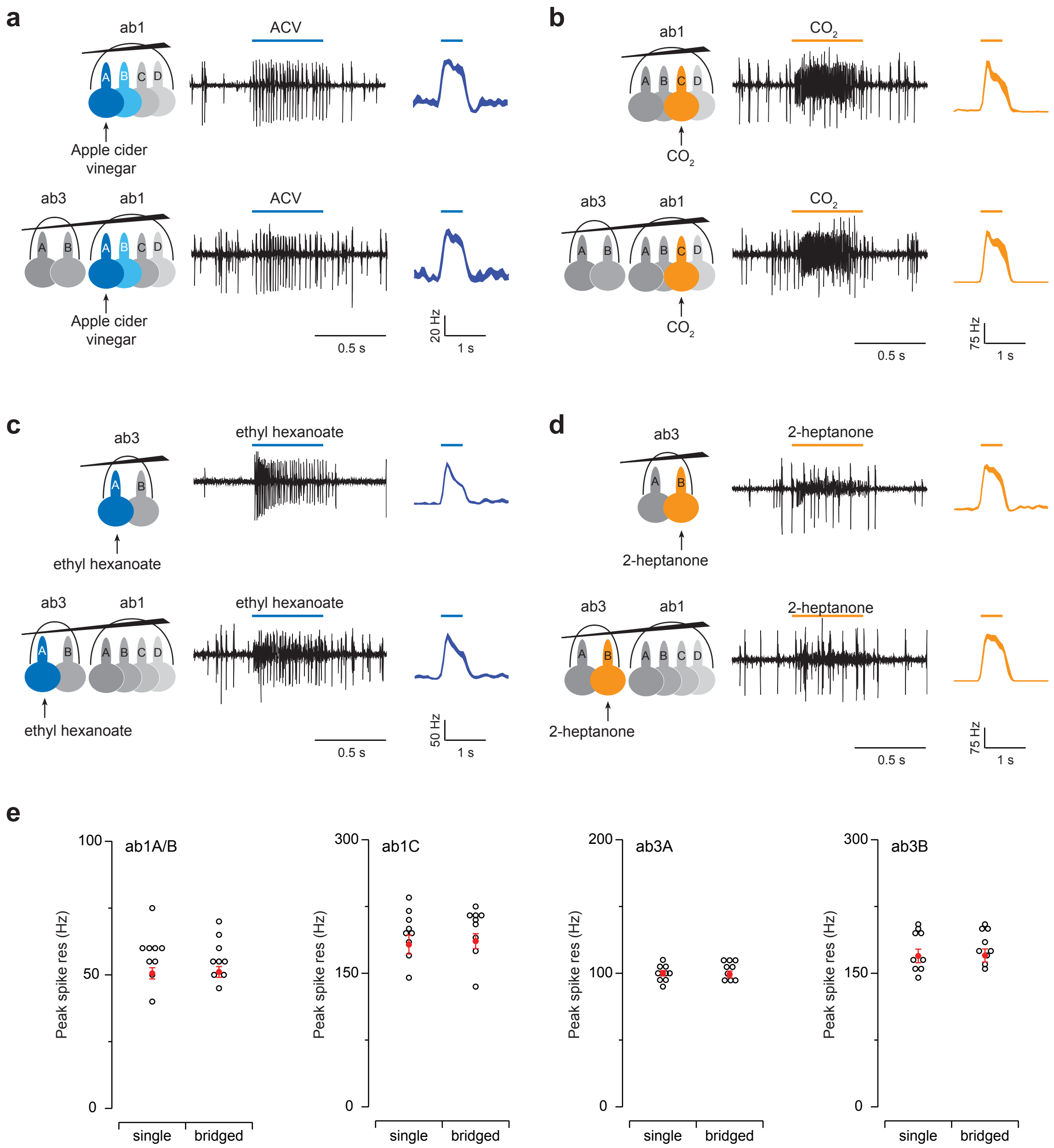

Supplementary Figure 1

Bridged recording configuration does not change ORN responses.

(a) ab1A/B responses to a pulse of apple cider vinegar (ACV, $3x10^{-6}$) were recorded using single-sensillum (top panel) or bridged two-sensillum recording configuration (bottom panel). ab1A and ab1B spikes could not be sorted reliably and were grouped. Distal sensillum: ab3; proximal sensillum: ab1.
(b) As in **(a)**, except that a pulse of CO2 (~1%) was used to activate ab1C.
(c-d) As in **(a-b)**, except that a pulse of ethyl hexanoate ($3x10^{-6}$) was used to activate ab3A **(c)** and a pulse of 2-heptanone ($3x10^{-6}$) was used to activate ab3B **(d)**. Of note, the spike amplitudes of ab3 ORNs were smaller when recorded using the bridged configuration in the distal sensillum than when recorded in the single-sensillum configuration.
(e) Quantification of the peak responses, mean ± s.e.m. ab1A/B, $P = 0.746$; ab1C, $P = 0.654$; ab3A, $P = 0.366$; ab3B, $P = 0.683$ (n=9), paired t-test.

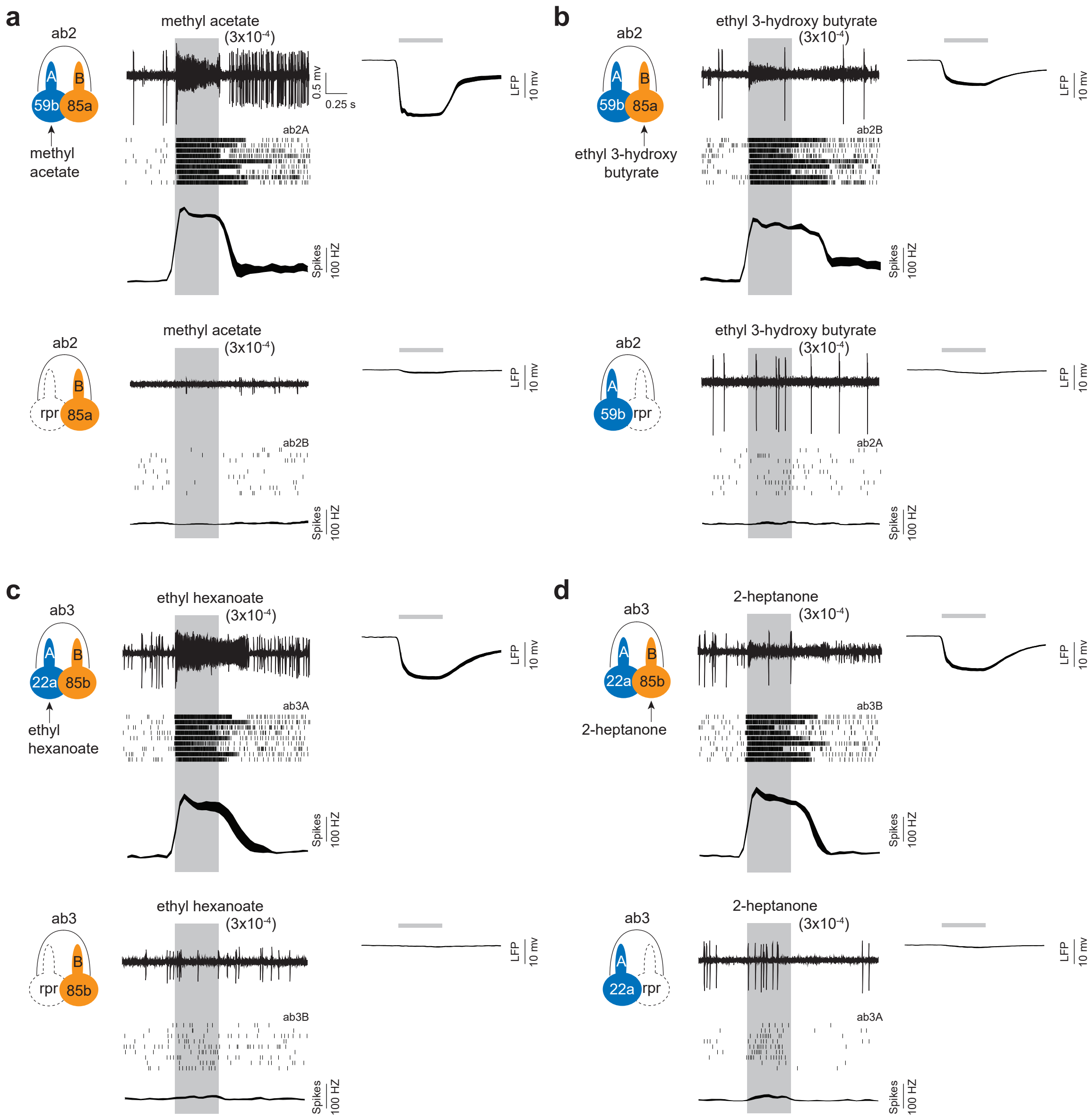

Supplementary Figure 2

Identification of private odorants for grouped ORNs.

Odorants from published datasets were selected to screen for private odorants[1-7]. **(a)** Top panels: ab2A is strongly activated by methyl acetate ($3x10^{-4}$ dilution). Sample trace for the spike response (top), ab2A raster plots (middle), and the average ab2A peri-stimulus time histogram (bottom) are shown. Upper right panel: the corresponding LFP response. Line width indicates s.e.m. Bottom panels: a cell death gene, reaper (rpr) is expressed in ab2A to selectively ablate the neuron. In the absence of ab2A, methyl acetate ($3x10^{-4}$) scarcely elicits any LFP response in the ab2 sensillum, indicating that methyl acetate-elicited LFP responses originate mainly from ab2A activation. *n=9*, mean ± s.e.m., parallel experiments.
(b) As in **(a)**, except that ab2B responses are shown. Ethyl 3-hydroxy butyrate is a private odorant for ab2B. Genetic ablation of ab2B abolishes the LFP response elicited by ethyl 3-hydroxy butyrate ($3x10^{-4}$ dilution) in the ab2 sensillum.
(c-d) Identification of private odorants for ab3A **(c)** and ab3B **(d)**. Single-sensillum recordings and genetic ablation experiments were carried out as described in **(a)**. Private odorants do not elicit significant LFP responses in the absence of the target ORNs. The odorants are not considered “private” above the indicated concentrations because they will also activate the neighboring neurons. *n=9*, mean ± s.e.m., parallel experiments. Similar genetic ablation experiments were conducted to identify private odorants for 7 additional pairs of grouped ORNs (see **Supplementary Table 1** for summary).

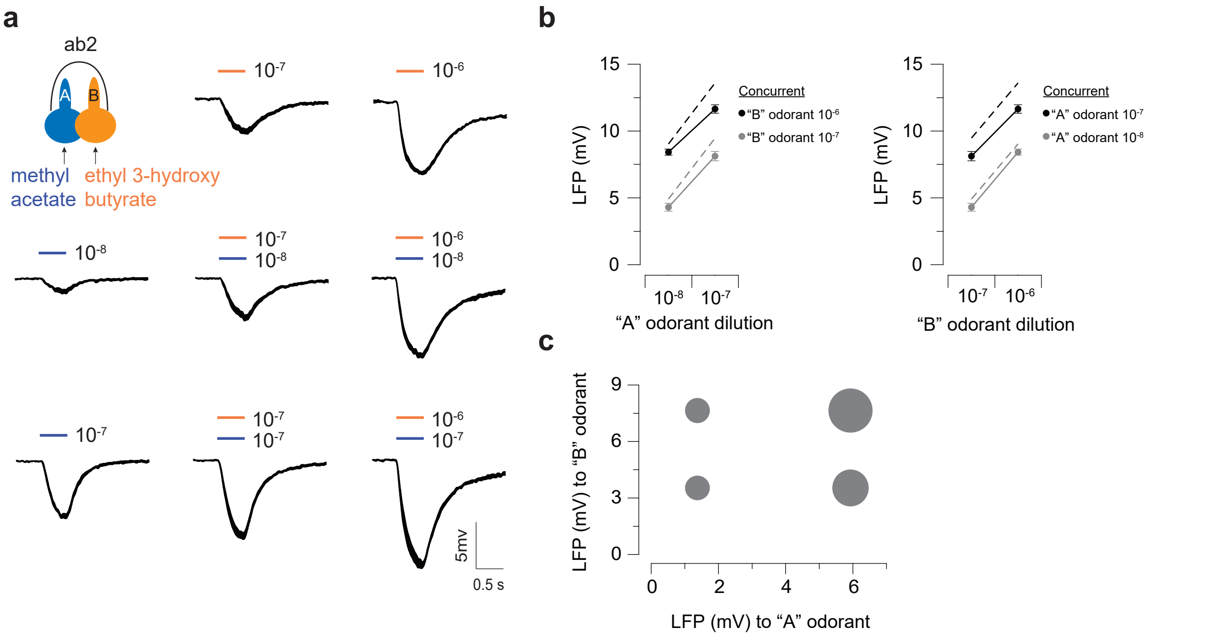

Supplementary Figure 3

The degree of ephaptic inhibition is influenced by the field responses of grouped ORNs.

(a) LFP responses of the ab2 ORNs to 0.5-sec pulses of their private odorants (ab2A: methyl acetate, blue bar; ab2B: ethyl 3-hydroxy butyrate, orange bar). Odorants were delivered either as individuals or as concurrent binary odor mixtures. *n=8* sensilla, line width indicates s.e.m.
(b) Peak LFP responses (absolute values) are plotted as a function of odorant dilution for methyl acetate (left panel) or ethyl 3-hydroxy butyrate (right panel). The concentrations of the concurrent odorants that activate the neighboring neurons are indicated. The linear sums of the LFP responses to individual private odorants are connected by dashed lines, predicting the LFP responses to odor mixtures if there is no ephaptic inhibition. The measured LFP responses were smaller than the linear sums, indicating ephaptic inhibition between ORNs, mean ± s.e.m.
(c) Bubble plot of the magnitude of inhibition in relation to the LFP responses to methyl acetate ("A" odorant, x-axis) or ethyl 3-hydroxy butyrate ("B" odorant, y-axis). Inhibition was determined by subtracting the measured LFP response from the linear sum. The size of the bubble scales with the magnitude of inhibition.

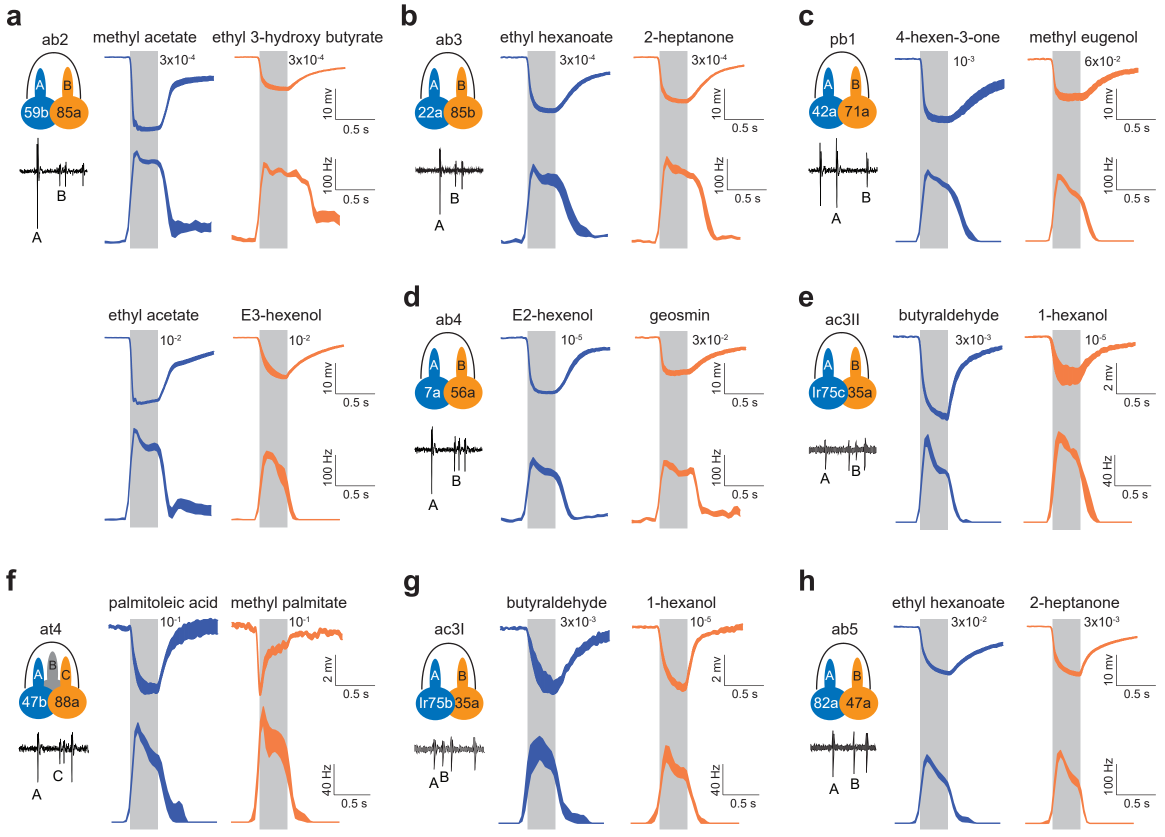

Supplementary Figure 4

LFP and the corresponding spike responses of grouped ORNs to private odorants.

ORNs were stimulated with their respective private odorants at the highest possible concentrations (see also **Supplementary Table 1**). Average LFP and the corresponding spike responses are shown for the large-spiking "A" neurons (blue traces) and the small-spiking neighbors (orange traces). Gray rectangles denote periods of odor stimulation (0.5 sec). The concentrations of each odorant are indicated. **(a-h)** Responses were recorded from neighboring neurons housed in the same sensillum. Eight sensillum types were examined (ab2, ab3, ab4, pb1, ac3 type I, ac3 type II, at4). *n=9* pairs of ORNs, except for ac3: *n=6* pairs, line width indicates s.e.m.

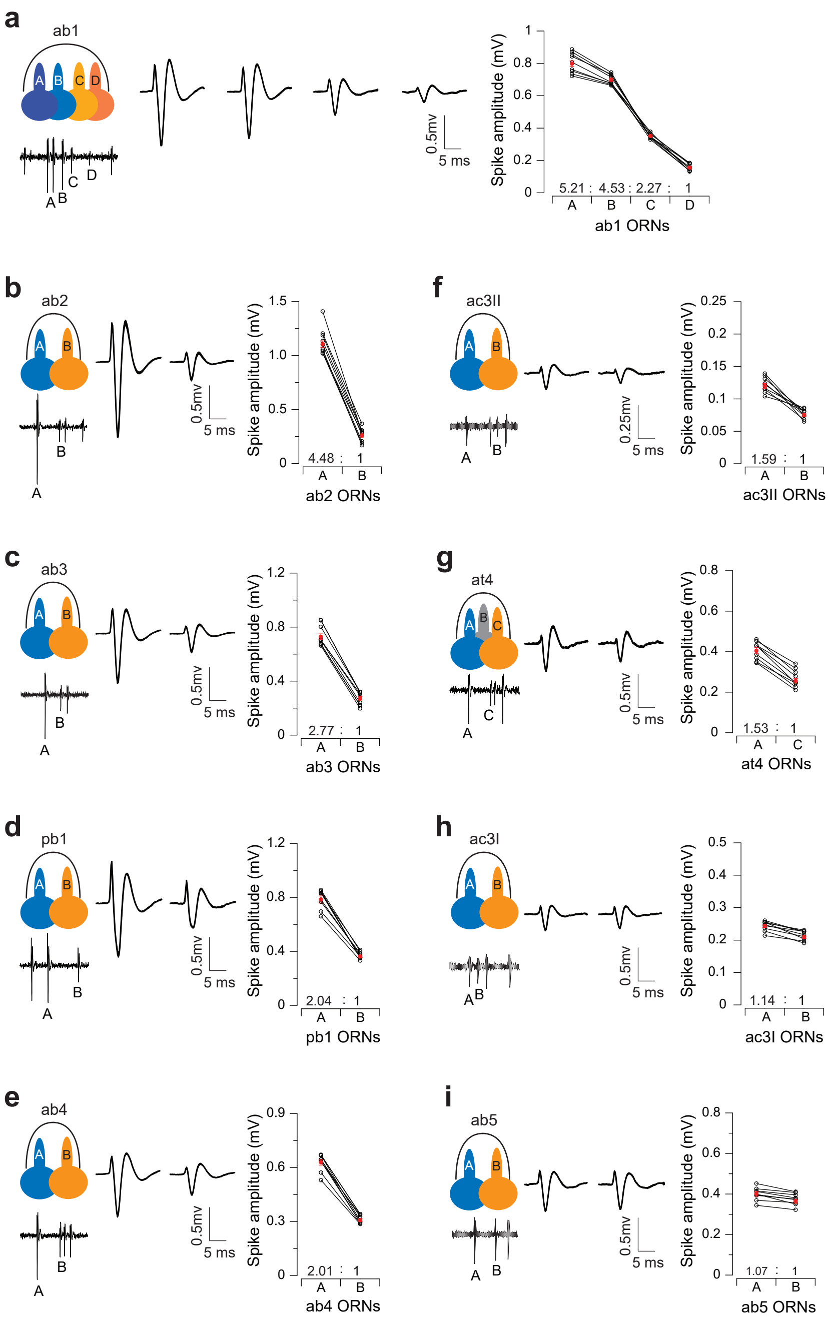

Supplementary Figure 5

Relative extracellular spike amplitudes of grouped ORNs.

Spontaneous spike activities from compartmentalized ORNs were recorded to characterize their extracellular spike amplitudes in nine different types of sensilla **(a-i)**. Recordings were bandpass filtered at 100-20k Hz. Left: Average spike waveforms are shown for each of the grouped ORNs. Line width indicates s.e.m. Right: Comparison of the spike amplitudes of compartmentalized ORNs. Each data point represents the average spike amplitude of an ORN based on its spontaneous activity. Lines connect measurements from the same recording. Red dots denote average spike amplitudes. Spike amplitude ratios, relative to the paired ORN with the smallest spike, are indicated for each sensillum types. *n=9*, mean ± s.e.m. Note: for technical issues, the signal-to-noise ratio for ac3 type II is lower than that for other sensillum types and the absolute spike amplitudes may thus be underestimated. See Online **Methods** for the identification of ac3 type I and type II sensilla.

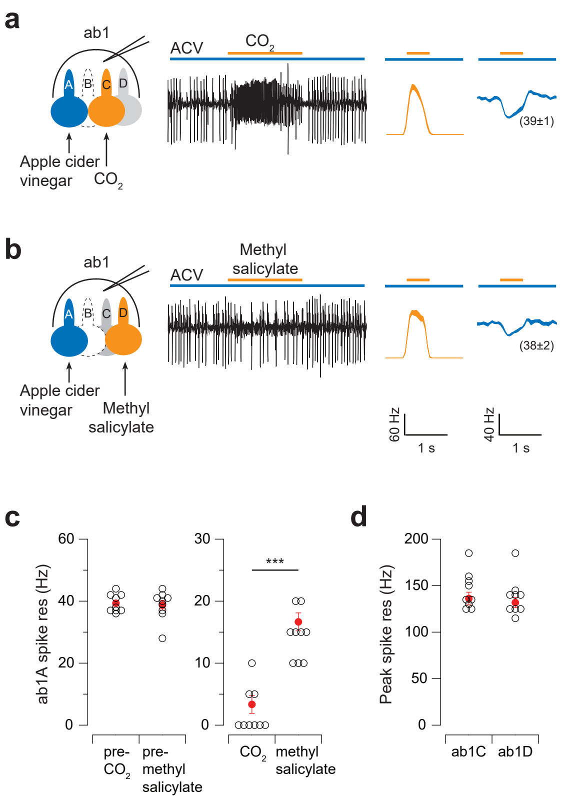

Supplementary Figure 6

ab1C is more effective than ab1D in inhibiting the chronic response of ab1A.

(a) The sustained response of ab1A was cross-inhibited by the transient activation of ab1C. The ab1B ORNs were ablated by the ectopic expression of a cell death gene, *rpr*. ab1A responded (large spikes in trace) to a sustained stimulus of apple cider vinegar (10^{-2} dilution, v/v in water, blue bar). A 500-ms pulse of CO_2 ($5x10^{-2}$ dilution, v/v in air, orange bar above trace) activated ab1C (small spikes). In the average spike responses on the right, the orange trace represents the response of ab1C to CO_2, and the blue trace represents ab1A response. The sustained spike response of ab1A (blue traces) is indicated in the parentheses (spikes sec^{-1}). Line width indicates s.e.m.
(b) As in **(a)**, except that ab1D in the same sensillum was activated by methyl salicylate (10^{-5} dilution, v/v in paraffin oil, orange bar above trace).
(c) Quantification of the ab1A spike responses before (left) and during CO_2 or methyl salicylate stimulation (right). Pre, P = 0.697; During, P = 0.00003 *(n=9)*, paired *t*-test.
(d) Quantification of the peak spike responses of ab1C and ab1D upon CO_2 or methyl salicylate stimulation. Mean ± s.e.m. P = 0.572 *(n=9)*, paired *t*-test.

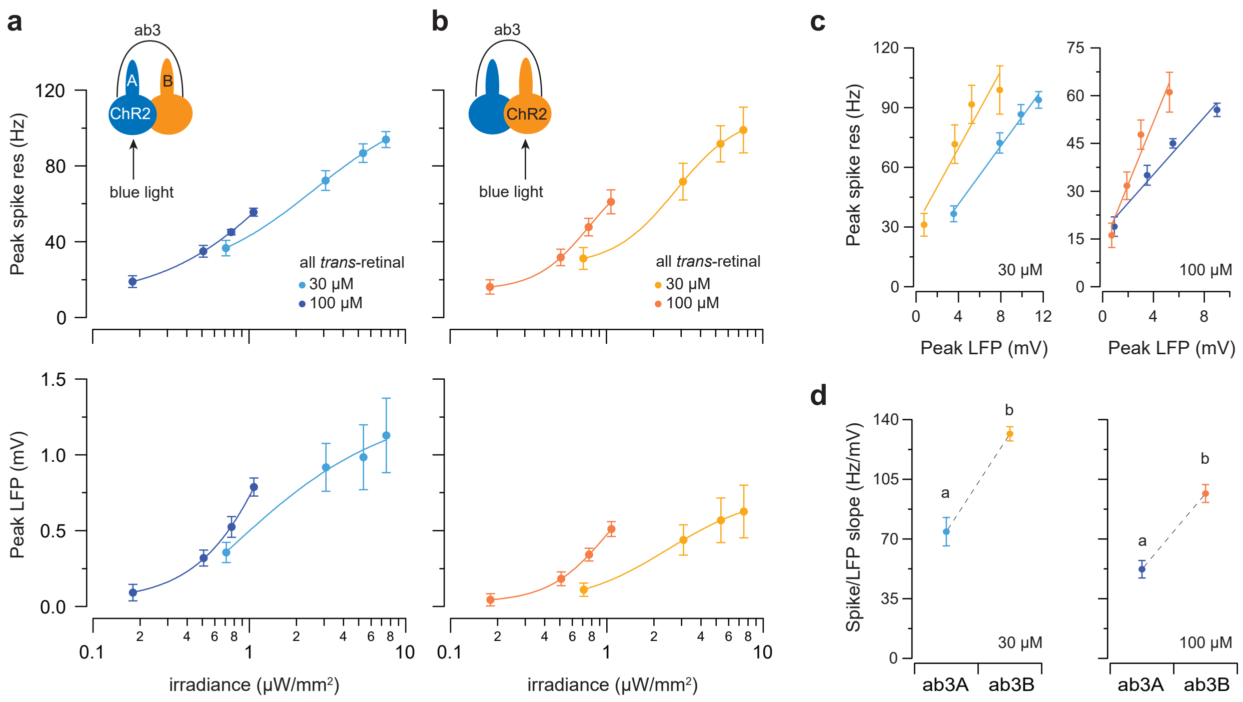

Supplementary Figure 7

Optogenetic analysis with different retinal concentrations.

(a-b) H134R-Channelrhodopsin2 (ChR2) was expressed in either ab3A **(a)** or ab3B **(b)** by the GAL4-UAS system. Newly emerged female flies were fed with the chromophore, all trans-retinal, at indicated concentrations (30 or 100 μM) for 5 days prior to experiments. ORNs were activated by 500-ms pulses of blue light of graded irradiances. Dose-response curves are shown to demonstrate the peak spike (top panels) and peak LFP responses (bottom panels). Results are from parallel experiments for each retinal concentration. *n=9*, mean ± s.e.m.
(c) Peak spike responses are plotted as a function of peak LFP responses. Lines indicate linear fits *(y = ax + b)*. The spike/LFP relationships are shown based on the retinal concentrations.
(d) The respective “a” coefficients (spike/LFP slope) for ab3A and ab3B are plotted for comparison. Dotted lines link results from parallel experiments. Statistical analysis was performed with ANCOVA and significant differences (*P* < 0.05) are denoted by different letters. Error bars = s.d. The P values are (c) 0.006 and (d) 0.005.

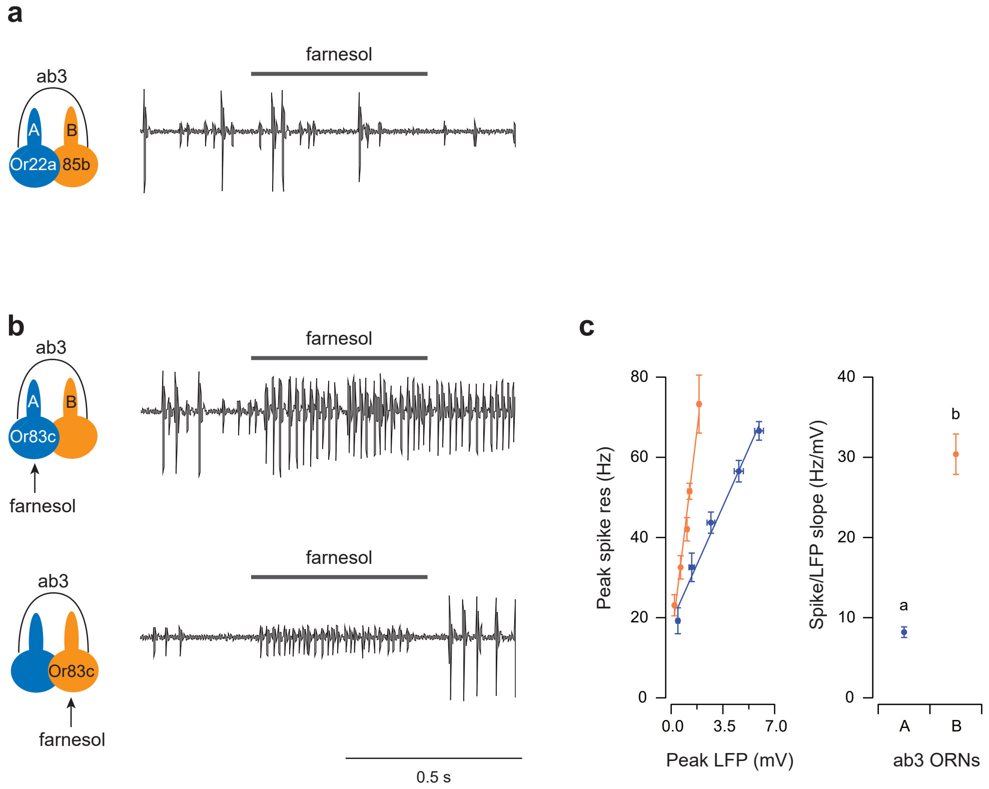

Supplementary Figure 8

Spike/LFP analysis of ab3 ORNs expressing the Or83c receptor.

(a) Farnesol at 2x10^{-2} dilution did not activate ab3A or ab3B ORNs in control flies.
(b) Or83c was ectopically expressed in either ab3A or ab3B using the GAL4-UAS system to confer responses to farnesol in the target ORNs.
(c) Peak spike responses are plotted as a function of peak LFP responses to different concentrations of farnesol. Left panel: Lines indicate linear fits ($y = ax + b$). n=9, mean ± s.e.m. Right panel: The respective "a" coefficients (spike/LFP slope) for ab3A and ab3B are plotted for comparison. Error bars = s.d. Statistical analysis was performed with ANCOVA and significant differences ($P < 0.05$) are denoted by different letters. The P value is 0.00012.

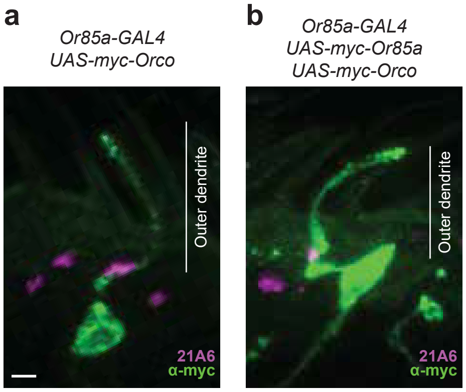

Supplementary Figure 9

Dendritic targeting of ectopically expressed odorant receptors in ORNs.

Confocal images of antennal sections immunolabeled with an anti-myc antibody (green) and an antibody specific for the sensory cilium base marker 21A6 (magenta). Ectopic expression of myc-tagged Orco **(a)** or myc-Orco and myc-Or85a **(b)** in the ab2B ORNs using Or85a-GAL4 driver. Immunofluorescence signal of myc labeling is observed throughout the target ORNs, including the sensory dendrites where olfactory transduction takes place. The anti-myc signal was stronger when myc-ORCO and myc-OR85a were co-expressed. Images in **(a)** and **(b)** were acquired in parallel with identical parameters. Scale bar, 2 μm.

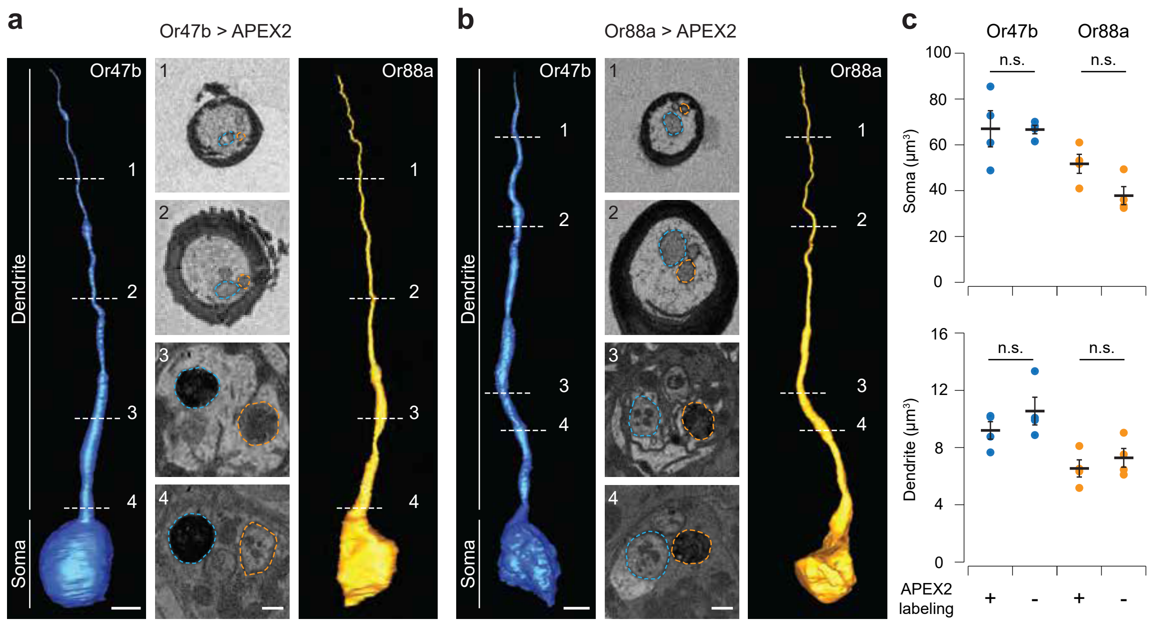

Supplementary Figure 10

Morphometric measurements of genetically identified ORNs with SBEM.

(a-b) 3D reconstruction of the Or47b (blue) and Or88a ORNs (orange) based on the SBEM volumes generated from Or47b>APEX2 and Or88a>APEX2 antenna, respectively.
(a) Or47b ORNs expressing APEX2 were labeled with DAB. Sample SBEM images are shown in the middle panel. Dotted lines outline the Or47b ORN (blue) and its intermediate-sized neighbor (orange).
(b) Or88a ORNs expressing APEX2 were labeled with DAB. In the sample SBEM images, dotted lines outline the Or88a ORN (orange) and its largest-sized neighbor (blue). Scale bars: 2 µm for 3D models, 500 nm for SBEM images.
(c) The soma and dendritic volumes of the Or47b and Or88a ORNs, mean ± s.e.m. *n*=4 pairs of ORNs. APEX2-labeled vs. unlabeled Or47b soma, *P* = 0.725; Or88a soma, *P* = 0.224; Or47b dendrite, *P* = 0.274; Or88a dendrite, *P* = 0.167, *t*-test.

Supplementary Table 1
Private odorants for select ORN pairs in *Drosophila* .

Sensillum	ORN Pair		Receptor	Private Odorant	Highest concentration used (v/v dilution in paraffin oil)	Peak spike response (Hz)
Large Basiconic	ab2	A	Or59b	methyl acetate	3.00E-04	295±4
		B	Or85a	ethyl 3-hydoxybutyrate	3.00E-05	253±8
	ab2	A	Or59b	ethyl acetate	1.00E-02	291±6
		B	Or85a	E3-hexenol	1.00E-02	210±11
	ab3	A	Or22a	ethyl hexanoate	3.00E-04	252±9
		B	Or85b	2-heptanone	3.00E-04	276±10
Small Basiconic	ab4	A	Or7a	E2-hexenal	1.00E-05	206±10
		B	Or56a	geosmin	3.00E-02	210±7
	ab5	A	Or82a	geranyl acetate	3.00E-02	212±9
		B	Or47a	pentyl acetate	3.00E-03	227±9
	pb1	A	Or42a	4-hexen-3-one	1.00E-03	226±6
		B	Or71a	methyl eugenol	6.00E-02	207±9
Coeloconic	ac3I	A	Ir75b	butyraldehyde	3.00E-03	105±8
		B	Or35a	1-hexanol	1.00E-05	109±7
	ac3II	A	Ir75c	butyraldehyde	3.00E-03	98±14
		B	Or35a	1-hexanol	1.00E-05	90±10
Trichoid	at4	A	Or47b	palmitoleic acid	1.00E-01	123±5
		C	Or88a	methyl palmitate	1.00E-01	138±10

Supplementary Table 2
Morphometric measurements of grouped ORNs.
The two at4 datasets were acquired independently based on the identity of APEX2-labeled ORN, as indicated by the asterisks. See Methods for details on quantifications. *n=4-5* per sensillum type.

ORN type	Soma volume (μm^3)	Soma surface area (μm^2)	Inner dendrite volume (μm^3)	Inner dendrite length (µm)	Outer dendrite volume (μm^3)	Outer dendrite length (µm)	Derived outer dendrite surface area (μm^2)
ab3A	47.2 ± 0.7	98.1 ± 3.8	13.4 ± 1.2	12.7 ± 0.9	n.a.	n.a.	n.a.
ab3B	31.2 ± 1.3	90.3 ± 4.4	4.1 ± 0.2	11.0 ± 1.0	n.a.	n.a.	n.a.
ab4A	51.0 ± 4.5	137.4 ± 11.3	5.8 ± 0.2	7.5 ± 1.2	n.a.	n.a.	n.a.
ab4B	33.4 ± 0.9	75.3 ± 0.7	2.4 ± 0.2	9.0 ± 0.9	n.a.	n.a.	n.a.
ab5A	30.2 ± 1.2	65.2 ± 3.3	3.6 ± 0.2	5.2 ± 0.4	0.7 ± 0.0	17.0 ± 0.4	19.9 ± 2.5
ab5B	38.2 ± 3.7	67.0 ± 4.1	1.7 ± 0.2	4.1 ± 0.4	0.7 ± 0.2	17.8 ± 1.2	19.7 ± 4.8
ac3IIA	40.7 ± 1.4	96.5 ± 3.6	3.6 ± 0.4	12.7 ± 1.3	0.9 ± 0.1	11.5 ± 0.4	11.8 ± 0.5
ac3IIB	23.7 ± 1.4	60.7 ± 3.9	2.2 ± 0.2	13.8 ± 1.7	0.6 ± 0.1	12.3 ± 1.0	10.3 ± 1.1
at4A*	67.0 ± 7.9	96.0 ± 7.8	7.5 ± 0.4	15.5 ± 1.5	1.7 ± 0.3	24.0 ± 1.8	22.5 ± 3.0
at4C	37.8 ± 3.9	73.0 ± 5.6	6.0 ± 0.4	17.1 ± 2.1	1.3 ± 0.4	24.4 ± 1.4	19.7 ± 2.9
at4A	66.7 ± 1.8	103.0 ± 2.4	6.8 ± 0.8	12.9 ± 1.9	3.7 ± 0.3	23.1 ± 0.9	36.8 ± 3.7
at4C*	51.7 ± 4.1	95.3 ± 0.8	5.6 ± 0.7	15.3 ± 2.2	0.9 ± 0.1	25.5 ± 0.7	19.1 ± 2.3

Supplementary Table 4
Fly genotypes.

Figure 1	a	Wild-type *Canton-S*
		Gr63a^{1} *(Bloomington #9941, RRID:BDSC_9941) (Jones et al., 2007)*
	b	Wild-type *Canton-S*
	c	*UAS-H134R-ChR2 (Bloomington #28995, RRID:BDSC_28995); Or22a-GAL4 (Bloomington #9951, RRID:BDSC_9951)*
Figure 2	a-h	Wild-type *Canton-S*
Figure 3	a-b	Wild-type *Canton-S*
	c-d	*UAS-H134R-ChR2; Or22a-GAL4 (Bloomington #9951, RRID:BDSC_9951)*
		UAS-H134R-ChR2; Or85b-GAL4 (Bloomington #23912, RRID:BDSC_23912)
	e	*UAS-H134R-ChR2; Or42b-GAL4 (Bloomington #9972, RRID:BDSC_9972)*
		UAS-H134R-ChR2; Or92a-GAL4 (Bloomington #23139, RRID:BDSC_23139)
		UAS-H134R-ChR2; Gr21a-GAL4 (Bloomington #23890, RRID:BDSC_23890)
		UAS-H134R-ChR2; Or10a-GAL4 (Bloomington #23885, RRID:BDSC_23885)
		UAS-H134R-ChR2; Or59b-GAL4 (Bloomington #23897, RRID:BDSC_23897)
		UAS-H134R-ChR2; Or85a-GAL4/Tm3-Sb
		UAS-H134R-ChR2; Or47b-GAL4 (Bloomington #9984, RRID:BDSC_9984) (Fishilevich and Vosshall, 2005)
		UAS-H134R-ChR2, Or88a-GAL4; + (Bloomington #23294, RRID:BDSC_23294)
		ΔOr7a^{GAL4} *(Lin et al, 2015); UAS-H134R-ChR2/Cyo; Dr/Tm3-Sb*
		UAS-H134R-ChR2; Or56a-GAL4 (Bloomington #23896, RRID:BDSC_23896)
		UAS-H134R-ChR2; Or42a-GAL4/Tm3-Sb (Bloomington #9969, RRID:BDSC_9969)
		UAS-H134R-ChR2; Or71a-GAL4/Tm3-Sb (Bloomington #23122, RRID:BDSC_23122)
Figure 4	a-b	*Or82a-GAL4/UAS-Or83c; + (Bloomington #23125, RRID:BDSC_23125) (Ronderos et al., 2014)*
		Or47a-GAL4/UAS-Or83c; +
	c-d	Wild-type *Canton-S*
	e-f	Wild-type *Canton-S*
Figure 5	a	*+; UAS-Or85a/+ (Bloomington #76051, RRID:BDSC_76051) (Hallem et al., 2004)*
		10xUAS-myc-Orco/+ (this study); Or85a-GAL4/UAS-Or85a (Bloomington #24461, RRID:BDSC_24461)
	b	*ΔOr7a*^{GAL4}*; Sp/Cyo; UAS-Or7a/Tm3-Sb (Bloomington #68454, RRID:BDSC_68454) (Hallem et al., 2004)*
		ΔOr7a^{GAL4}*; Sp/Cyo; UAS-Or85a/Tm3-Sb*
Figure 6	a	*10X UAS-mCD8GFP-APEX2 (Tsang et al., 2018); Or22a-GAL4*
	b	*10X UAS-myc-APEX2-Orco (Tsang et al., 2018); Or56a-GAL4*
	c	*10X UAS-myc-APEX2-Orco; Or47b-GAL4*
		10X UAS-myc-APEX2-Orco, Or88a-GAL4; +
	d	*10X UAS-myc-APEX2-Orco; Ir75c-GAL4 (Prieto-Godino et al., 2017)*
	e	*10X UAS-myc-APEX2-Orco; Or47a-GAL4 (Bloomington #9981, RRID:BDSC_9981)*
Figure S1	a-d	Wild-type *Canton-S*
Figure S2	a	Wild-type *Canton-S*
		UAS-rpr (Yao et al., 2005); Or59b-GAL4
	b	Wild-type *Canton-S*
		UAS-rpr; Or85a-GAL4
	c	Wild-type *Canton-S*
		UAS-rpr; Or22a-GAL4
	d	Wild-type *Canton-S*
		UAS-rpr; Or85b-GAL4
Figure S3	a-c	Wild-type *Canton-S*
Figure S4	a-h	Wild-type *Canton-S*
Figure S5	a-i	Wild-type *Canton-S*
Figure S6	a-d	*UAS-rpr; Or92a-GAL4*
Figure S7	a	*UAS-H134R-ChR2; Or22a-GAL4*
	b	*UAS-H134R-ChR2; Or85b-GAL4*
Figure S8	a	Wild-type *w*^{1118}
	b	*UAS-Or83c/+; Or22a-GAL4/+*
	c	*UAS-Or83c/+; Or85b-GAL4/+*
Figure S9	a	*10xUAS-myc-Orco/+; Or85a-GAL4/+*
	b	*10xUAS-myc-Orco/+; Or85a-GAL4/UAS-Or85a*
Figure S10	a	*10X UAS-cmyc-APEX2-Orco (Tsang et al., 2018); Or47b-GAL4*
	b	*10X UAS-cmyc-APEX2-Orco, Or88a-GAL4; +*
Table S2	ab3	*10X UAS-mCD8GFP-APEX2; Or22a-GAL4*
	ab4	*10X UAS-cmyc-APEX2-Orco; Or56a-GAL4*
	ab5	*10X UAS-cmyc-APEX2-Orco; Or47a-GAL4*
	ac3II	*10X UAS-cmyc-APEX2-Orco; Ir75c-GAL4*
	at4	*10X UAS-cmyc-APEX2-Orco; Or47b-GAL4 (at4A labeled)*
		10X UAS-cmyc-APEX2-Orco, Or88a-GAL4; + (at4C labeled)

Supplementary Table 5
Parameters for SBEM acquisitions.

sensilla type	dwell time (μsec)	condenser aperture (μm)	accelerating voltage (kV)	z step size (nm)	raster size	z dimension (# of sections)	chamber pressure in variable pressure mode (Pa)	pixel size (nm)	gas injection %
ab3	0.5	30	3	30	16k x 12k	1000	30	3.124	n.a.
ab4	1.5	30	2.5	40	14k x 11k	1423	not in variable pressure mode	5.96	85%
ab5	1	30	2	40	14k x 16k	999	not in variable pressure mode	4.379	40%
ac3II	1.5	30	2.5	50	12k x 16k	658	not in variable pressure mode	5.06	70%
at4 (Or47b labeled)	1	30	2.5	50	12k x 9k	1200	not in variable pressure mode	6.5	85%
at4 (Or88a labeled)	1.5	30	2.5	50	12k x 9k	1444	not in variable pressure mode	6.48	85%

References

1. Hallem, E. A. & Carlson, J. R. Coding of odors by a receptor repertoire. *Cell* **125,** 143–60 (2006).

2. Prieto-Godino, L. L. *et al.* Evolution of acid-sensing olfactory circuits in Drosophilids. *Neuron* **93,** 661–676 (2017).

3. Stensmyr, M. C. *et al.* A conserved dedicated olfactory circuit for detecting harmful microbes in Drosophila. *Cell* **151,** 1345–57 (2012).

4. Yao, C. A., Ignell, R. & Carlson, J. R. Chemosensory coding by neurons in the coeloconic sensilla of the Drosophila antenna. *J. Neurosci.* **25,** 8359–67 (2005).

5. Silbering, A. F. *et al.* Complementary function and integrated wiring of the evolutionarily distinct Drosophila olfactory subsystems. *J. Neurosci.* **31,** 13357–13375 (2011).

6. Dweck, H. K. M. *et al.* Pheromones mediating copulation and attraction in Drosophila. *Proc. Natl. Acad. Sci.* **112,** 2829–2835 (2015).

7. Lin, H.-H. *et al.* Hormonal modulation of pheromone detection enhances male courtship success. *Neuron* **90,** 1272–1285 (2016).

8. Maarse, H. *Volatile Compounds in Foods and Beverages*. (Marcel Dekker, 1991).

9. Chen, H.-C., Sheu, M.-J. & Wu, C.-M. Characterization of volatiles in guava (Psidium guajava L. cv. Chung-Shan Yueh-Pa) fruit from Taiwan. *J.Food Drug Anal.* **14,** 398–402 (2006).

10. Chyau, C. C., Chen, S. Y. & Wu, C. M. Differences of Volatile and Nonvolatile Constituents between Mature and Ripe Guava (Psidium Guajava Linn.) Fruits. *J. Agric. Food Chem.* **40,** 846–849 (1992).

11. Jordan, M. J., Goodner, K. & Shaw, P. E. Volatile components in banana (Musa acuminata colla cv. cavendish) and yellow passion fruit (Passiflora edulis Sims. f. flavicarpa Degner) as determined by GC-MS and GC-olfactometry. *Florida State Hortic. Soc.* **114,** 153–157 (2001).

12. Macoris, M. S., Janzantti, N. S., Garruti, D. dos S. & Monteiro, M. Volatile compounds from organic and conventional passion fruit (Passiflora edulis F. Flavicarpa) pulp. *Ciência e Tecnol. Aliment.* **31,** 430–435 (2011).

13. Pello-Palma, J. *et al.* Determination of volatile compounds in cider apple juices using a covalently bonded ionic liquid coating as the stationary phase in gas chromatography. *Anal. Bioanal. Chem.* **409,** 3033–3041 (2017).

14. Pinu, F. & Villas-boas, S. G. Rapid Quantification of Major Volatile Metabolites in Fermented Food and Beverages Using Gas Chromatography-Mass Spectrometry. *Metabolites* **7,** 37 (2017).

15. Qian, K., Zhu, J. J., Sims, S. R., Taylor, D. B. & Zeng, X. Identification of volatile compounds from a food-grade vinegar attractive to house flies (Diptera: Muscidae). *J. Econ. Entomol.* **106,** 979–987 (2013).

16. Stensmyr, M. C., Larsson, M. C., Bice, S. & Hansson, B. S. Detection of fruit- and flower-emitted volatiles by olfactory receptor neurons in the polyphagous fruit chafer Pachnoda marginata (Coleoptera: Cetoniinae). *J. Comp. Physiol. A.* **187,** 509–519 (2001).

17. Torrens, J., Riu-Aumatell, M., López-Tamames, E. & Buxaderas, S. Volatile compounds of red and white wines by headspace– solid-phase microextraction using different fibers. *J. Chromatogr. Sci.* **42,** 310–316 (2004).

18. Xu, Y., Fan, W. & Qian, M. C. Characterization of aroma compounds in apple cider using solvent-assisted flavor evaporation and headspace solid-phase microextraction. *J. Agric. Food Chem.* **55,** 3051–3057 (2007).
